# Annotations

**Published:** 1899-02-18

**Authors:** 


### Annotations.

### Out-Patient Practice.

The general acceptance of modern views as to the necessity of hygienic treatment for the cure of consumption must sooner or later raise the question how far physicians are morally justified in lending themselves to the ordinary routine of out-patient practice, and in thus giving their sanction to those methods of treatment by aid of drugs alone which are there so much in vogue. The position occupied by the hospitals in the eyes of the public is the outcome of the splendid results obtained among the in-patients in the wards. It is well recognised that by dint of the organisation and appliances which the hospitals possess, and in consequence of the individual care which is exercised by the medical staff in the treatment of each patient admitted to the wards, what is called "hospital treatment" is something superior to, and certainly something quite different from, what can be obtained by application to the "pay doctor." And this is recognised by the staff just as much as it is by the patients. Everyone knows that what is done to the in-patients is not a mere giving of drugs, but a treatment in the true sense of the word; and one of the great charms of hospital work undoubtedly lies in the fact that, unfettered by surroundings, one is able to put forth one's full powers for the benefit of one's patients. How different all is in the out-patient department! We are not going to raise again the old cry about the rapidity with which out-patients are seen by the physicians. What we would point out is quite another aspect of the question, namely, that the physician can but rarely do for his patient what he knows to be the best. If it be held that a man has done well when he has done his best "under the circumstances," then the out-patient physician no doubt does well when he sends his patient away with hope and a bottle of physic. But if by lending their sanction and by giving their services to great out-patient departments they are merely maintaining and keeping on its feet a system of drug-treatment which they know not to be the best, then it is open to suggest that it is time to reconsider their position. At a recent public meeting, Dr. Heron is reported as follows: "Speaking with a full sense of the responsibility he incurred, he maintained that the present system of the treatment of consumption in the out-patient departments of the hospitals at our large cities was little less than a farce. Just now some thousands of consumptives were attending the waiting-rooms of London hospitals. They might wait there for a long time before they could be seen, and when they were, what happened? The consumptive saw the physician for a minute or so, perhaps; the physician prescribed some drug for his patient, then with his bottle of medicine the consumptive went back to live, perhaps to starve, in a slum. To call this the proper treatment of consumption was a cruel mockery. The charitable public supplied the funds which supported hospitals, and it was for them to say how much longer they would permit such a waste of money and of time on this burlesque of charity." But surely these words do not apply to consumption alone! It is a serious matter that the hospitals and their staffs should throw all their influence into the scale in favour of a "bottle of physic treatment" if that sort of treatment is not the best. It is not only a wrong to the patients, but it acts injuriously upon the profession. How far the sanction given by the hospitals to the demand for a "bottle of physic" as a necessary part of treatment tends to demoralise both patients and physicians can only be understood by those who are conversant with the inner life of the profession. It may, however, be illustrated by a quotation from a letter in the *British Medical Journal* for last week, in which a well-qualified medical man, writing on an interesting subject in the most perfect good faith, and without the slightest suspicion that he is saying anything which might call for adverse comment, says, in describing his treatment of a case: "I gave no medicine whatever, except a little coloured water to please them." Here we see the true outcome of the teaching the students receive in the out-patient room. "A little coloured water to please them," and this said without the slightest idea that such a prescription suggests anything derogatory to the honour of the profession!

### The Feeding of the Children.

We are all agreed that not only is improper and deficient feeding the cause of an immense number of deaths among infants, but that it is also responsible for the puny stature and the stunted minds of many who grow up only to be an expense to the community of which they form a part. We need hardly be surprised, then, to find that a claim is being put in for the feeding of the infants at the public expense. Of course, it is rank socialism; of course, improvident parents who marry young, or even without marrying cast these infants upon the world, are bad, wicked, heartless, and everything that is evil; but still, there the children are, and the question is, What is to be done with them? If left alone unhelped, it is clear that in many cases they must either serve merely to swell the infant death-rate, or must grow up puny and deformed, a ready prey to disease and vice, and a curse to the community; and in view of the free gift of education which at enormous expense is forced upon a not altogether willing people, simply because it has been decided that it is good policy that children should be educated, it is not altogether unnatural that people should be

**ANNOTATIONS**—*continued.*

found to ask, Is it not good also that the children should be fed? And would it not be better to spend public money for a short time during infancy in securing that they shall grow up strong and straight and fit to earn a living, rather than to spend money in their support during those long years in after life when, in consequence of their imperfect development, they have become inmates of workhouses and reformatories and jails? Shall we, then, give free food as well as free education; in other words, shall we subsidise poor mothers to help them to bring up their offspring as valuable citizens instead of as "wasters?" It is an anxious question, and one of the very next that will have to come forward. With the tremendous birth-rate of the Anglo-Saxon race, children have, so far, been of small account. But the birth-rate is diminishing, and the market value of children ought to rise; and after all, a child, more than any animal that comes into the world, ought to be worth the rearing. Crowded although the population is, each good worker leaves the world a little richer; while each child spoilt in the rearing drags down the rest. And, so far as economics are concerned, that is the bottom of the question.

### The Prince of Wales and a Noble Profession.

THE visit of the Prince of Wales to the Royal College of Surgeons for the purpose of attending the Hunterian oration is but another proof of the honourable position which is now accorded to the art of healing. That the heir to the throne should himself be a Fellow of the Royal College of Physicians, and that this should be the third occasion on which he has been present at the great annual function held at the Royal College of Surgeons, shows an interest in all that pertains to the relief of suffering which is as honourable to the Prince himself as it is pregnant with honour to the profession which devotes itself to that work. Indeed it would be well to insist on this point, that it is because medicine is engaged in relieving suffering, and because, as Professor Allbutt has lately said, "the ideal side of the physician's life is that he brings healing or solace to his human fellow," that medicine can really claim the title of a noble profession. We fear, however, that the kind things which it is nowadays fashionable to say about leading medical men are to be taken more as a testimonial to their utility than to their humanity. It is only in accordance with the drift of the age—this utilitarian age, which measures everything by the uses to which it can be put—that honour should be given to medicine in proportion as it displays new powers of tinkering up the carnal body in which we live. It is no doubt a great and a wonderful thing, and, as honours go in this world, a thing worthy of the highest honour that, as a result of our art, we should be able, as the saying is, to "save life" to the extent that is now being done. On every side we come across instances of our skill. Men who would have died from the pressure of tumours on their brains are walking about, thinking their more or less crooked thoughts, to their own gratification, although not perhaps greatly to the advantage of the world; women who would have died from cancer or ovarian disease are now proud mothers of families, to whom they will doubtless transmit their own morbid tendencies; thousands of crooked-legged, ricketty, and scrofulous children are restored, or, at any rate, are made to walk, much to their own joy, but to the deterioration of the race; even in the very birth children are saved and mothers are saved who but for our efforts would have died; and all this life-saving is put down to our credit, notwithstanding that it does but perpetuate evils which Nature—that hard mother—would have eliminated by destroying all such imperfect individuals. This generation, however, cares little for posterity; each individual wants to live and get his pleasures out of life; that profession which tinkers and patches up his frame, which oils his creaky joints for him and makes him run a little longer, is clearly a profession of great utility to the individualist; and if it is honoured chiefly for what it does, we must accept the homage and neither wonder nor complain. This, however, is not the only nor the chief glory of medicine.

### Home Work and the Factory Acts.

Is it not time that the provisions of the Factory Acts should be extended to all places where work is done for money? At present any employer who has work done on his own premises is compelled to keep them in a clean and sanitary condition, and to limit the hours of any women and young persons whom he may employ. But, when work is given out, who can tell the conditions under which it is done? Such work being, as a rule, but poorly paid, the workers are for the most part women, some of whom, indeed, regard it only as a bye-work, by means of which they procure some luxuries which their ordinary income could not furnish. The homes of these are often good enough, and, from the point of view of the consumer, not much objection need be taken to what they produce. But there are others, working 12 or 14 hours a day to make from five to seven shillings a week, who, because they have to work so long to earn so little, have no time to attend to the cleanliness of their homes. They have no time to attend properly to their children, and these may languish and fall ill, perhaps of some infectious disease, without their mother paying much heed. They are too poor to spend much on furnishing, even to replace the things that were pawned when work was slack the last time, and the shirts, or aprons, or children's suits they are making come in usefully to eke out the scanty bed-clothes. The worker cannot take time to think of the possible consequences of this. But the purchaser of these garments might with advantage give a little thought to the possible consequences in their own families of buying and wearing clothes that have lain above a patient suffering from some form of infectious disease. It is difficult to abolish home-work, and certainly to do so would entail the complete pauperisation of many who now earn a scanty, but honourable, living at it. But surely it is not impossible to extend the Factory and Workshop Acts so that every place where a garment is made for sale should be licensed as a factory, even though no paid assistants are employed in it. We cannot control the conditions under which people make their own clothes, but we might try to look after those under which they make other people's.

